# Anticancer Effect of Ursodeoxycholic Acid in Human Oral Squamous Carcinoma HSC-3 Cells through the Caspases

**DOI:** 10.3390/nu7053200

**Published:** 2015-05-05

**Authors:** Liang Pang, Xin Zhao, Weiwei Liu, Jiang Deng, Xiaotong Tan, Lihua Qiu

**Affiliations:** 1 Department of Oral and Maxillofacial Surgery, Stomatological Hospital of Chongqing Medical University, Chongqing 401147, China; E-Mails: pangliang@foods.ac.cn (L.P.); dengjiang@foods.ac.cn (J.D.); 2 Chongqing Key Laboratory for Oral Diseases and Biomedical Sciences, Chongqing 401147, China; 3 Department of Biological and Chemical Engineering, Chongqing University of Education, Chongqing 400067, China; E-Mail: zhaoxin@cque.edu.cn; 4 Institute of Functional Ecological Food, Chongqing University of Education, Chongqing 400067, China; 5 School of Public Health and Management, Chongqing Medical University, Chongqing 400016, China; E-Mail: weiweiliu521@gmail.com; 6 Department of Food Science and Nutrition, Pusan National University, Busan 609-735, Korea; E-Mail: tanxiaotong@pusan.ac.kr; 7 Chongqing Municipal Key Laboratory of Oral Biomedical Engineering of Higher Education, Chongqing 401147, China

**Keywords:** ursodeoxycholic acid, human oral squamous carcinoma HSC-3 cells, apoptosis, caspase, expression

## Abstract

Bear bile was used as a traditional medicine or tonic in East Asia, and ursodeoxycholic acid (UDCA) is the most important compound in bear bile. Further, synthetic UDCA is also used in modern medicine and nutrition; therefore, its further functional effects warrant research, *in vitro* methods could be used for the fundamental research of its anticancer effects. In this study, the apoptotic effects of UDCA in human oral squamous carcinoma HSC-3 cells through the activation of caspases were observed by the experimental methods of MTT (3-(4,5-dimethyl-2-thiazolyl)-2,5-diphenyl-2-*H*-tetrazolium bromide) assay, DAPI (4’,6-diamidino-2-phenylindole) staining, flow cytometry analysis, RT-PCR (reverse transcription-polymerase chain reaction) assay and Western blot assay after HSC-3 cells were treated by different concentrations of UDCA. With 0 to 400 μg/mL UDCA treatment, UDCA had strong growth inhibitory effects in HSC-3 cells, but had almost no effect in HOK normal oral cells. At concentrations of 100, 200 and 400 μg/mL, UDCA could induce apoptosis compared to untreated control HSC-3 cells. Treatment of 400 μg/mL UDCA could induce more apoptotic cancer cells than 100 and 200 μg/mL treatment; the sub-G1 DNA content of 400 μg/mL UDCA treated cancer cells was 41.3% *versus* 10.6% (100 μg/mL) and 22.4% (200 μg/mL). After different concentrations of UDCA treatment, the mRNA and protein expressions of caspase-3, caspase-8, caspase-9, Bax, Fas/FasL (Fas ligand), TRAIL (TNF-related apoptosis-inducing ligand), DR4 (death receptor 4) and DR5 (death receptor 5) were increased in HSC-3 cells, and mRNA and protein expressions of Bcl-2 (B-cell lymphoma 2), Bcl-xL (B-cell lymphoma-extra large), XIAP (X-linked inhibitor of apoptosis protein), cIAP-1 (cellular inhibitor of apoptosis 1), cIAP-2 (cellular inhibitor of apoptosis 2) and survival were decreased. Meanwhile, at the highest concentration of 400 μg/mL, caspase-3, caspase-8, caspase-9, Bax, Fas/FasL, TRAIL, DR4, DR5, and IκB-α expression levels were the highest, and Bcl-2, Bcl-xL, XIAP, cIAP-1, cIAP-2, survival, and NF-κB expression levels were the lowest. These results proved that UDCA could induce apoptosis of HSC-3 cancer cells through caspase activation, and the higher concentration of UDCA had stronger effects *in vitro*. UDCA might be a good nutrient for oral cancer prevention.

## 1. Introduction

In traditional Asian medicine, bear bile extracted from the gallbladder of *Ursus thibetanus* or *Ursus arctos* is considered a cure for various diseases. With the development of modern medicine, it is found that the major active component in bear bile is ursodeoxycholic acid (UDCA) [1]. Nowadays, synthetic UDCA is a safe drug with no side effects and is widely used in the treatment of diseases, such as gallstones, primary cirrhosis, autoimmune hepatitis and colon cancer around the world [2].

Oral cancers are common malignant tumors in the head and neck and are mostly categorized as squamous cell carcinomas, in which the mucous membranes in the gum, tongue, and face transform and progress to cancer [3]. There are many causes of oral cancers, including poor oral hygiene caused by bad living habits and chronic ulcers in gum tissue. Due to the long-term intake of a high-fat, high-protein, and low-cellulose diet, each organ in the digestive system functions abnormally, producing a large amount of harmful bacteria leading to a large number of harmful bacteria growing in the mouth, which produce carcinogens that cause oral cancers [4]. Chronic inflammation, from, for example, gingivitis and periodontitis, may be another cause of oral cancer [5]. Written in ancient Chinese medical books, bear bile can treat bacterial diseases through sterilization. Laboratory experiments further confirmed that the main component of bear bile, UDCA, has the effect of sterilization and inhibition of inflammation [1]. Bamboo salt UDCA toothpastes appear on the market in China and South Korea, and clinical surveys prove that UDCA can prevent gum and periodontal problems and reduce gum bleeding. Therefore, UDCA may be useful in treating oral cancers.

Inducing cancer cell apoptosis is one way to control cancer, so it is beneficial for the treatment of oral cancers to increase activation, expression, and regulation of caspase family genes leading to death of oral cancer cells [6]. Although the detailed mechanism of apoptosis is not entirely clear, it has been identified that caspases play an indispensable role in apoptosis. The apoptotic process is actually the cascading amplification of caspase hydrolysis of their substrates [7]. Using UDCA to strengthen caspase activity may be a new way for the treatment of oral cancers by inducing apoptosis of oral cancer cells. In this study, through using UDCA treatment *in vitro* on cultured cancer cells, changes in caspase gene expression of cancer cells are detected and the effect of UDCA on inducing cancer cell apoptosis is observed.

## 2. Materials and Methods

### 2.1. Preparations of Ursodeoxycholic Acid (UDCA)

UDCA monomer was purchased from Shanghai Ekear Biotechnology Co. Ltd. (Shanghai, China), chenodeoxycholic acid (CDCA) monomer was purchased from Nanjing Senbeijia Biological Technology Co. Ltd. (Nanjing, China) and deoxycholic acid (DCA) monomer was purchased from Shanghai Pureone Biotechnology Co. Ltd. (Shanghai, China).

### 2.2. Cell Preparation

Human oral squamous carcinoma HSC-3 cells were obtained from American Type Culture Collection (Manassas, VA, USA), TCA8113 human tongue carcinoma cells obtained from Shanghai Institute of Biochemistry and Cell Biology (Shanghai, China) and HOK normal oral epithelial cells were obtained from ScienCell Research Laboratories (Carlsbad, CA, USA). These cells were cultured in DMEM medium (Thermo Fisher Scientific Inc.; Waltham, MA, USA) supplemented with 10% fetal bovine serum (FBS) and 1% penicillin-streptomycin (Gibco-BRL; Grand Island, NY, USA) at 37 °C in a humidified atmosphere with 5% CO_2_ (incubator model 311 S/N29035; Forma, Waltham, MA, USA). The medium was changed 2–3 times a week [8].

### 2.3. 3-(4,5-Dimethyl-2-Thiazolyl)-2,5-Diphenyltetrazolium Bromide (MTT) Assay

Growth of HSC-3, TCA8113 and HOK cells after UDCA, CDCA and DCA treatment were assessed by the MTT assay. UDCA, CDCA and DCA were added to the DMEM medium, the UDCA, CDCA and DCA culture medium solutions were made at concentrations of 0–500 μg/mL. HSC-3 and HOK cells were seeded in 96 well plates at a density of 1 × 10^5^ cells/mL in each well (100 μL) and incubated at 37 °C in 5% CO_2_. After 24 h culture, the culture medium was aspirated and discarded from each well, then the UDCA, CDCA and DCA containing culture medium was added to each well, then incubated for 48 h. The culture medium was aspirated and discarded in each well again, then the 5 mg/mL MTT solution (Amresco; Solon, OH, USA) was added in each well (100 μL) and the cells were cultured for 4 h. Following removal of the MTT solution, 100 μL DMSO was added to each well and mixed for 30 min. Subsequently, the absorbance of each well was measured with an enzyme-linked immunosorbant assay (ELISA) reader (model 680; Bio-Rad; Hercules, CA, USA) at 490 nm [9].

### 2.4. Nuclear Staining with 4’,6-Diamidino-2-Phenylindole (DAPI)

HSC-3 cell solution (2 mL) was added to a 10 cm culture dish and incubated at 37 °C in 5% CO_2_ for 24 h. Then the culture medium was aspirated and discarded, followed by addition of the UDCA culture medium solutions (100, 200 and 400 μg/mL) and cultured for 48 h. UDCA untreated control cells and cells treated with the UDCA were harvested, washed with PBS twice, and fixed with 3.7% paraformaldehyde (Sigma; St. Louis, MO, USA) in PBS for 10 min at 25 °C. The fixed cells were washed with PBS and stained with 1 mg/mL DAPI (Sigma) solution for 10 min. The cells were washed 3 times with PBS and examined with a fluorescence microscope (BX50; Olympus, Tokyo, Japan) [10].

### 2.5. Flow Cytometry Analysis

After treatment with DAPI for the first 72 h, the cells were trypsinized, collected, washed with cold PBS, and resuspended in 2 mL PBS. DNA content of the cells were measured using a DNA staining kit (CycleTESTTM PLUS kit; Becton Dickinson, Franklin Lakes, NJ, USA). Nuclear fractions stained with propidium iodide were obtained by following the manufacturer’s protocol. Fluorescence intensity was determined using the FACScan flow cytometer (EPICS XL-MCL; Beckman Coulter KK, Brea, CA, USA) and analyzed with CellQuest software (Becton Dickinson) [7].

### 2.6. RT-PCR Assay

Total RNA from HSC-3 cells was isolated using Trizol reagent (Invitrogen; Carlsbad, CA, USA) according to the manufacturer’s recommendations. The cancer cell RNA was digested with RNase-free DNase (Roche; Basel, Switzerland) for 15 min at 37 °C and purified using the RNeasy kit (Qiagen; Hilden, Germany) according to the manufacturer’s protocol. cDNA was synthesized from 2 μg of total RNA by incubation at 37 °C for l h with avian myeloblastosis virus reverse transcriptase (GE Healthcare; Little Chalfont, United Kingdom) with random hexanucleotides according to the manufacturer’s instruction. Sequences of primers used to specifically amplify the genes of interest are shown in Table 1. Amplification was performed in a thermal cycler (Eppendorf; Hamburg, Germany). The polymerase chain reaction (PCR) products were separated in 1.0% agarose gels and visualized with ethidium bromide staining [11].

**Table 1:** Sequences of reverse transcription-polymerase chain reaction primers were used in this study.

Gene Name	Sequence
Caspase-3	Forward: 5′-CAA ACT TTT TCA GAG GGG ATC G-3′
	Reverse: 5′-GCA TAC TGT TTC AGC ATG GCA-3′
Caspase-8	Forward: 5′-CCC CAC CCT CAC TTT GCT-3′
	Reverse: 5′-GGA GGA CCA GGC TCA CTT A-3′
Caspase-9	Forward: 5′-GGC CCT TCC TCG CTT CAT CTC-3′
	Reverse: 5′-GGT CCT TGG GCC TTC CTG GTA T-3′
Bax	Forward: 5′-AAG CTG AGC GAG TGT CTC CGG CG-3′
	Reverse: 5′-CAG ATG CCG GTT CAG GTA CTC AGT C-3′
Bcl-2	Forward: 5′-CTC GTC GCT ACC GTC GTG ACT TGG-3′
	Reverse: 5′-CAG ATG CCG GTT CAG GTA CTC AGT C-3′
Bcl-xL	Forward: 5′-CCC AGA AAG GAT ACA GCT GG-3′
	Reverse: 5′-GCG ATC CGA CTC ACC AAT AC-3′
XIAP	Forward: 5′-CCG TGC GGT TGC TTT AGT TGT C-3′
	Reverse: 5′-ATG GCA GGG TTC CTC GGG TAT-3′
cIAP-1	Forward: 5′-TGAGCATGCAGACACATGC-3′
	Reverse: 5′-TGACGGATGAACTCCTGTCC-3′
cIAP-2	Forward: 5′-AATGGAAGATAGCACGAT-3′
	Reverse: 5′-AGAAAGGCTGGAGTAAGA-3′
Survivin	Forward: 5′-CTT TCT CAA GGC CCA CCG CAT CT-3′
	Reverse: 5′-GCA CTT TCT CCG CAG TTT CCT C-3′
Fas	Forward: 5′-GAA ATG AAA TCC AAA GCT-3′
	Reverse: 5′-TAA TTT AGA GGC AAA GTG GC-3′
FasL	Forward: 5′-GGA TTG GGC CTG GGG ATG TTT CA-3′
	Reverse: 5′-TTG TGG CTC AGG GGC AGG TTG TTG-3′
TRAIL	Forward: 5′-GGA ACC CAA GGT GGG TAG AT-3′
	Reverse: 5′-TCT CAC CAC ACT GCA ACC TC-3′
DR4	Forward: 5′-AAG TCC CTG CAC CAC GAC-3′
	Reverse: 5′-CCA CAA CCT GAG CCG ATG-3′
DR5	Forward: 5′-TGA GAT AAA GGT GGC TAA A -3′
	Reverse: 5′-AAA GGT AAA CCA GGG AAG -3′
NF-κB	Forward: 5′-CAC TTA TGG ACA ACT ATG AGG TCT CTG G-3′
	Reverse: 5′-CTG TCT TGT GGA CAA CGC AGT GGA ATT TTA GG-3′
IκB-α	Forward: 5′-GCT GAA GAA GGA GCG GCT ACT-3′
	Reverse: 5′-TCG TAC TCC TCG TCT TTC ATG GA-3′
GAPDH	Forward: 5′-CGG AGT CAA CGG ATT TGG TC-3′
	Reverse: 5′-AGC CTT CTC CAT GGT CGT GA-3′

### 2.7. Western Blot Assay

Total protein was obtained from the HSC-3 cells using Radio-Immunoprecipitation assay buffer as previously described [12]. The protein concentrations were determined using a Bio-Rad protein assay kit. The nitrocellulose membranes (Schleicher and Schuell; Keene, NH, USA) were then subjected to immunoblot analysis and the proteins were visualized using an enhanced chemiluminescence (ECL) method (GE Healthcare). Fifty micrograms of protein from treated cells was loaded onto the gels. The cell lysates were separated using 12% SDS-PAGE, transferred onto a polyvinylidene fluoride membrane (GE Healthcare), blocked with 5% skim milk and then hybridized with primary antibodies (diluted 1:1000). The antibodies against caspase-3, caspase-8, caspase-9, Bax, Bcl-2, Bcl-xL, XIAP, cIAP-1, cIAP-2, survivin, Fas, FasL, TRAIL, DR4, DR5, NF-κB and IκB-α were obtained from Santa Cruz Biotechnology (Santa Cruz, CA, USA). The membranes were then incubated with the HRP-conjugated secondary antibodies (Santa Cruz Biotechnology) for 1 h at room temperature. The blots were washed 3 times with PBS-T and then developed using an ECL reagent (Amersham Life Science; Arlington Heights, IL, USA).

### 2.8. Statistical Analysis

Data are presented as mean ± standard deviation (SD). Differences between the mean values for individual groups were assessed with one-way analysis of variance (ANOVA) with Student-Neumann-Keuls *post-hoc* test, and the data were tested for a two-sided statistical testing. *p* < 0.01 and *p* < 0.05 were considered to indicate a statistically significant difference. SPSS software 19.0 (IBM Software; New York, NY, USA) was used for statistical analyses.

## 3. Results

### 3.1. Inhibitory Effects of Ursodeoxycholic Acid (UDCA), Chenodeoxycholic Acid (CDCA) and Deoxycholic Acid (DCA) on Cell Growth

**Figure 1:**
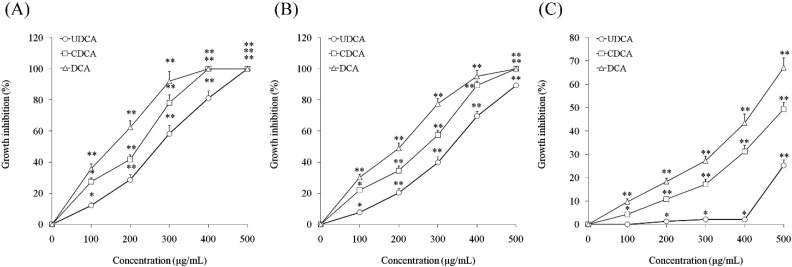
Growth inhibitory effects of ursodeoxycholic acid (UDCA), chenodeoxycholic acid (CDCA) and deoxycholic acid (DCA) in human oral squamous carcinoma HSC-3 cells (**A**), TCA8113 human tongue carcinoma cells (**B**), and HOK normal oral epithelial cells (**C**). * Mean values are significantly different from 0 μg/mL sample treatment (*p* < 0.05) and ** mean values are significantly different from 0 μg/mL sample treatment (*p* < 0.01).

*In vitro* growth effects of UDCA, CDCA and DCA on oral cancer and normal cells were evaluated using the MTT assay. At concentrations ranging from 0 to 500 μg/mL of UDCA, CDCA and DCA, human oral squamous carcinoma HSC-3 cells and TCA8113 cell viabilities were decreased by UDCA, CDCA and DCA in a concentration-dependent manner. At a concentration of 500 μg/mL, inhibition of the HSC-3 cells treated with UDCA reached 100% and at a concentration of 400 μg/mL, inhibition of the HSC-3 cells treated with CDCA and DCA reached 100% (Figure 1A). UDCA, CDCA and DCA also showed similar inhibitory effects in HSC-3 cells and TCA8113 cells at 0 to 500 μg/mL (Figure 1B). HOK normal oral epithelial cells were also evaluated, and there was no response in HOK normal oral cells treated with UDCA at concentrations of 0–400 μg/mL, but inhibition started once the concentration of UDCA exceeded 400 μg/mL (Figure 1C). HOK normal cell growth was also decreased by CDCA and DCA in a concentration-dependent manner. The inhibitory rates were higher than UDCA treatment from 0–500 μg/mL. At 0–400 μg/mL, UDCA had no toxic effect in normal oral cells, and only showed a lethal effect in oral cancer cells. This might be an apoptotic effect. CDCA and DCA showed toxic effects in normal cells at concentrations from 0 to 400 μg/mL. From these results, UDCA as a bile acid, it was different from CDCA and DCA. Consequently, concentrations of 100, 200 and 400 μg/mL were selected for subsequent experiments.

### 3.2. Induction of Apoptosis by UDCA

**Figure 2:**
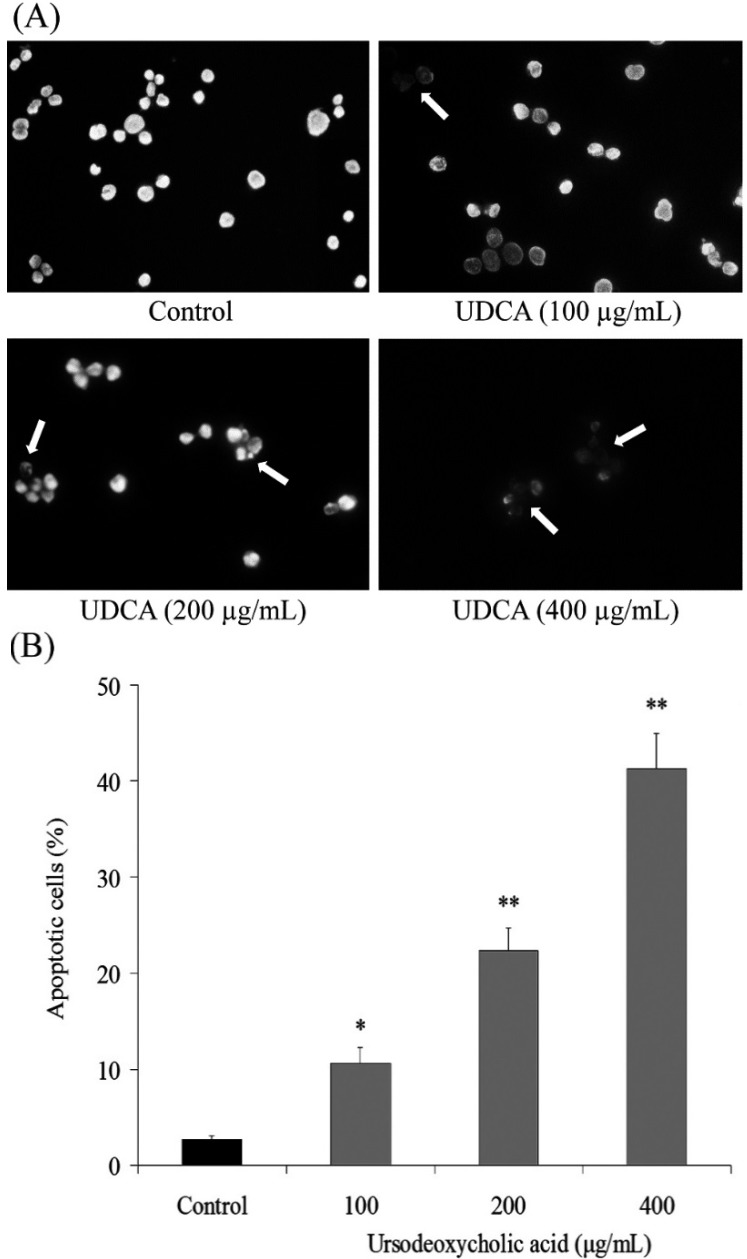
Exposure of human oral squamous carcinoma HSC-3 cells to ursodeoxycholic acid (UDCA) induced apoptosis. (**A**) Appearance of apoptotic bodies in HSC-3 cells treated with UDCA for 48 h (200×); and (**B**) treatment with UDCA for 48 h increased the number of apoptotic cells as measured by flow cytometry. The profile represents an increased sub-G1 population (apoptotic cells). * Mean values are significantly different from control (*p* < 0.05) and ** mean values are significantly different from control (*p* < 0.01).

To determine a possible mechanism underlying the growth inhibitory activity of UDCA in HSC-3 oral cancer cells, the induction of apoptosis was explored. The extent of chromatin condensation was analyzed by fluorescence microscopy of cells stained with the DNA-binding fluorescent dye DAPI and flow cytometric analysis. While the untreated HSC-3 cancer cells presented nuclei with homogeneous chromatin distribution, treatment with UDCA induced chromatin condensation and nuclear fragmentation, which suggests initiation of apoptosis. At a high concentration of 400 μg/mL, the UDCA treated HSC-3 cells underwent apoptosis and only a small amount of HSC-3 cancer cells were not affected. (Figure 2A). Chromatin condensation and the formation of apoptotic bodies, two hallmarks of apoptosis, were observed in cells cultured with 200 and 400 μg/mL of UDCA. In contrast, the level of chromatin condensation was low in the untreated control cells. Flow cytometric analyses revealed that treatment with UDCA promoted apoptosis of the HSC-3 cells compared with incubation with control cells. This conclusion is based on the significant accumulation of 100, 200 and 400 μg/mL treated cancer cells with sub-G1 DNA content at 10.6%, 22.4% and 41.3% compared to the control cells (2.7%) (Figure 2B).

### 3.3. Gene Expression of Caspase Family 

**Figure 3:**
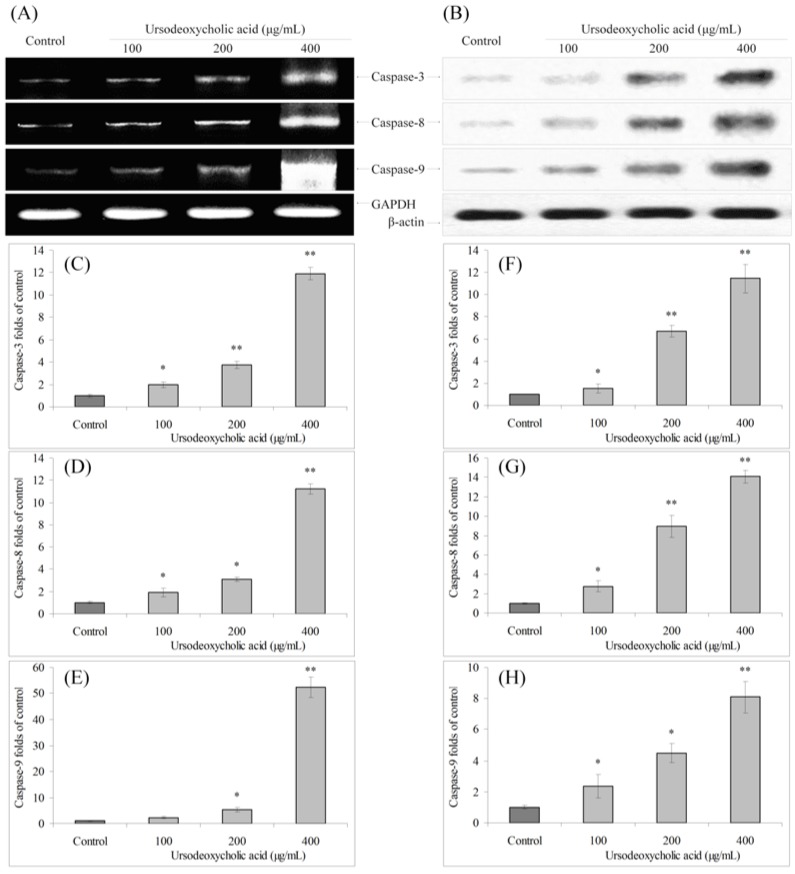
Effects of ursodeoxycholic acid (UDCA) on the mRNA (**A**) and protein (**B**) expression of capase-3, capase-8 and capase-9 in human oral squamous carcinoma HSC-3 cells. Fold-ratio: gene expression/GAPDH (β-actin) × control numerical value (control fold ratio: 1); (**C**), caspase-3 mRNA expression fold over control; (**D**), caspase-8 mRNA expression fold over control; (**E**), caspase-9 mRNA expression fold over control; (**F**), caspase-3 protein expression fold over control; (**G**), caspase-8 protein expression fold over control; and (**H**), caspase-9 protein expression fold over control. * Mean values are significantly different from control (*p* < 0.05) and ** mean values are significantly different from control (*p* < 0.01).

The mRNA expression of caspase-3, caspase-8 and caspase-9 in HSC-3 oral cancer cells were analyzed by RT-PCR and Western blot assays. After UDCA treatment, the mRNA and protein expression of caspase-3, caspase-8 and caspase-9 were increased compare to the control cells, and these changes were increased in a concentration-dependent manner (Figure 3).

### 3.4. Gene Expression of Bcl-2 Family

**Figure 4:**
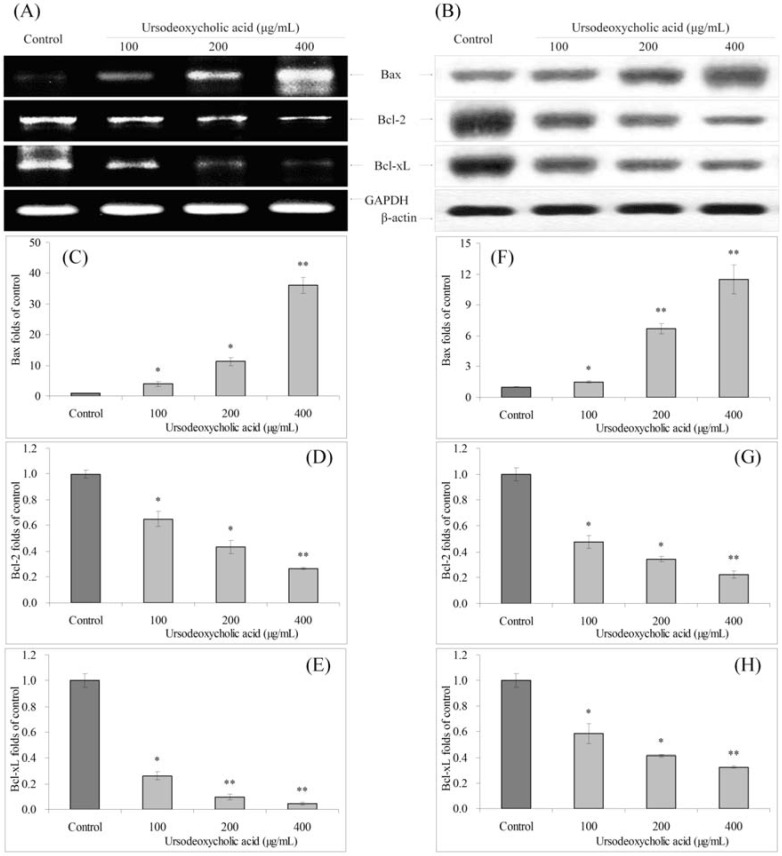
Effects of ursodeoxycholic acid (UDCA) on the mRNA (**A**) and protein (**B**) expression of Bax, Bcl-2 and Bcl-xL in human oral squamous carcinoma HSC-3 cells. Fold-ratio: gene expression/GAPDH (β-actin) × control numerical value (control fold ratio: 1); (**C**), Bax mRNA expression fold over control; (**D**), Bcl-2 mRNA expression fold over control; (**E**), Bcl-xL mRNA expression fold over control; (**F**), Bax protein expression fold over control; (**G**), Bcl-2 protein expression fold over control; and (**H**), Bcl-xL protein expression fold over control. * Mean values are significantly different from control (*p* < 0.05) and ** mean values are significantly different from control (*p* < 0.01).

UDCA also significantly changed the mRNA and protein expression of Bax, Bcl-2 and Bcl-xL in HSC-3 cancer cells (Figure 4). At the concentration of 400 μg/mL, UDCA treated cancer cells showed the highest Bax expression, and lowest Bcl-2, Bcl-xL expressions. The Bax expressions of 100 and 200 μg/mL UDCA treated cancer cells were also higher than control cells, and Bcl-2, Bcl-xL expressions were lower than those of control cells.

### 3.5. Gene Expression of XIAP, cIAP-1, cIAP-2 and Survivin

**Figure 5:**
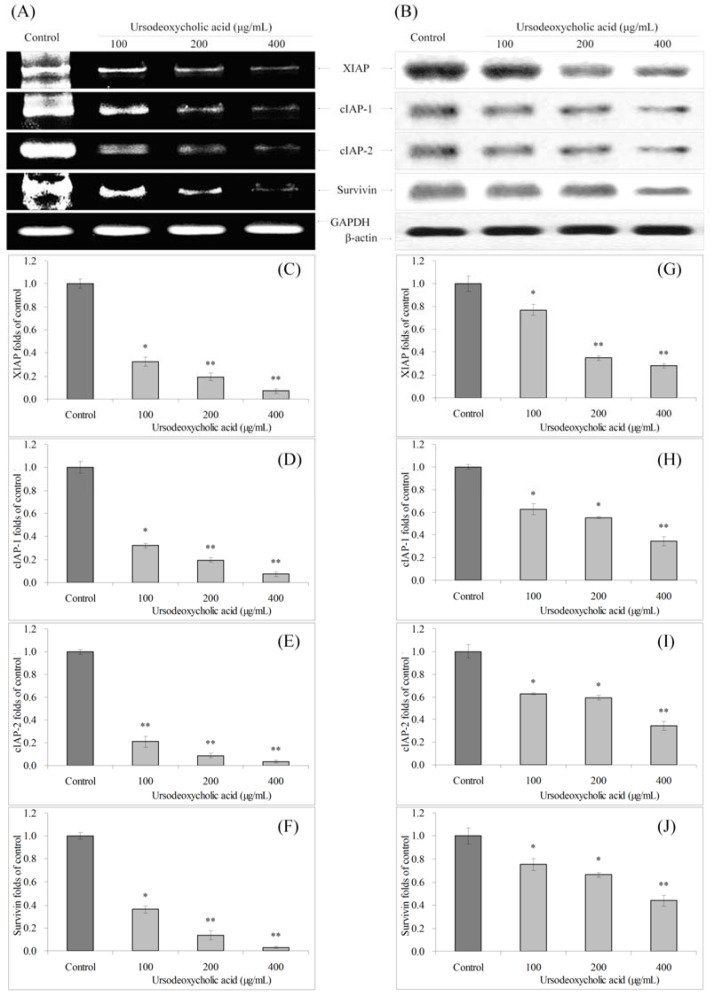
Effects of ursodeoxycholic acid (UDCA) on the mRNA (**A**) and protein (**B**) expression of XIAP, cIAP-1, cIAP-2 and survivin in human oral squamous carcinoma HSC-3 cells. Fold-ratio: gene expression/GAPDH (β-actin) × control numerical value (control fold ratio: 1); (**C**), XIAP mRNA expression folds of control; (**D**), cIAP-1 mRNA expression folds of control; (**E**), cIAP-2 mRNA expression folds of control; (**F**), survivin mRNA expression folds of control; (**G**), XIAP protein expression folds of control; (**H**), cIAP-1 protein expression folds of control; (**I**), cIAP-2 protein expression folds of control; and (**J**), survivin protein expression folds of control. * Mean values are significantly different from control (*p* < 0.05) and ** mean values are significantly different from control (*p* < 0.01).

The mRNA and protein expressions of XIAP, cIAP-1, cIAP-2 and survivin were reduced in UDCA treated HSC-3 cancer cells (Figure 5). The 100, 200 and 400 μg/mL UDCA treated cells had lower XIAP, cIAP-1, cIAP-2 and survivin expression than control HSC-3 cells.

### 3.6. Gene Expression of Fas and FasL

**Figure 6:**
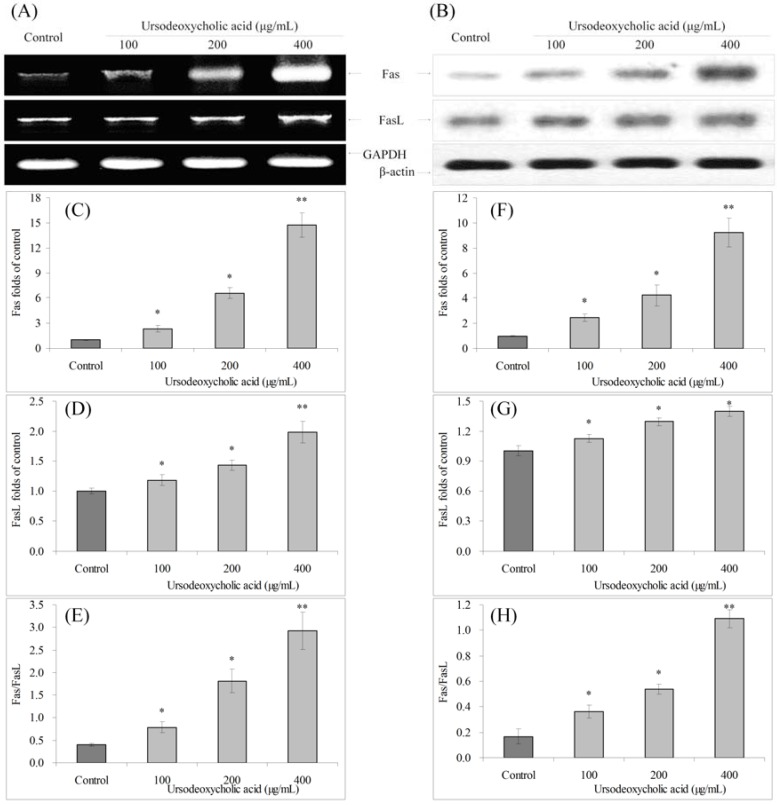
Effects of ursodeoxycholic acid (UDCA) on the mRNA (**A**) and protein (**B**) expression of Fas and FasL in human oral squamous carcinoma HSC-3 cells. Fold-ratio: gene expression/GAPDH (β-actin) × control numerical value (control fold ratio: 1); (**C**), Fas mRNA expression folds of control; (**D**), FasL mRNA expression folds of control; (**E**), Fas/FasL mRNA expression proportion; (**F**), Fas protein expression folds of control; (**G**), FasL protein expression folds of control; and (**H**), Fas/FasL protein expression proportion. * Mean values are significantly different from control (*p* < 0.05) and ** mean values are significantly different from control (*p* < 0.01).

Fas and FasL mRNA and protein expressions were increased, and the Fas/FasL ratio was also increased with UDCA treatment. Fas expression increased after UDCA treatment, and FasL expression increased slightly (Figure 6). The Fas/FasL ratio with 400 μg/mL UDCA treated cancer cells was much higher than control cells.

### 3.7. Gene Expression of TRAIL, DR4 and DR5

**Figure 7:**
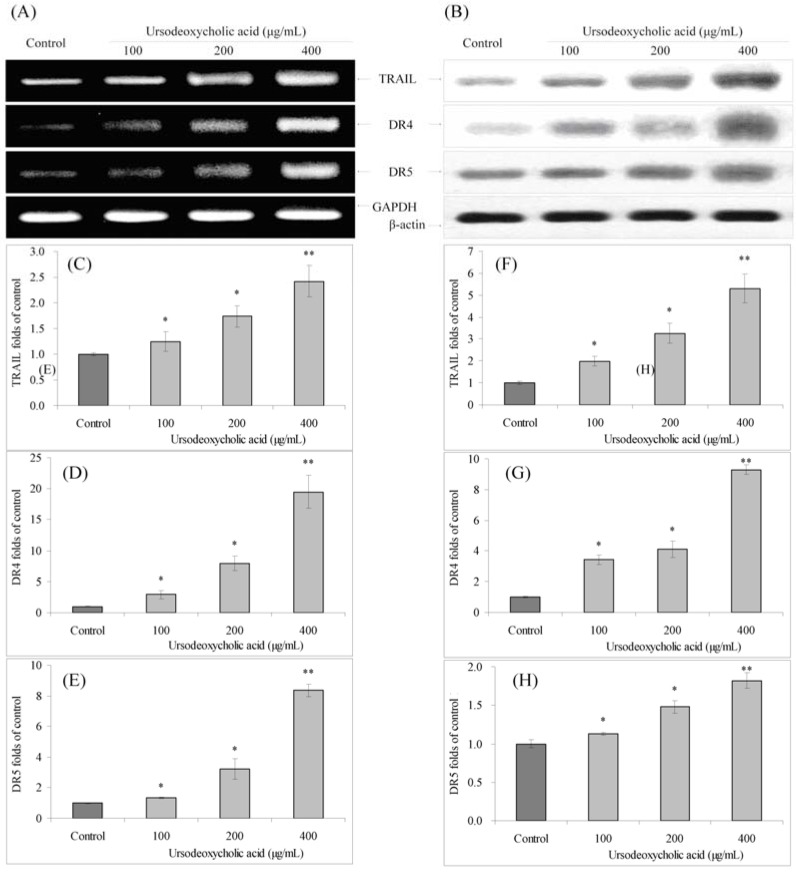
Effects of ursodeoxycholic acid (UDCA) on the mRNA (**A**) and protein (**B**) expression of TRAIL, DR4 and DR5 in human oral squamous carcinoma HSC-3 cells. Fold-ratio: gene expression/GAPDH (β-actin) × control numerical value (control fold ratio: 1); (**C**), TRAIL mRNA expression folds of control; (**D**), DR4 mRNA expression folds of control; (**E**), DR5 mRNA expression folds of control; (**F**), TRAIL protein expression folds of control; (**G**), DR4 protein expression folds of control; and (**H**), DR5 protein expression folds of control. * Mean values are significantly different from control (*p* < 0.05) and ** mean values are significantly different from control (*p* < 0.01).

UDCA also significantly increased the mRNA and protein expression of TRAIL, DR4 and DR5 compared to control cells (Figure 7). UDCA increased the expression of DR4 and DR5, with the mRNA expression with 400 μg/mL UDCA treatment were 19.46 and 8.34 times greater, and protein expression were 9.31 and 1.82 times greater than control cells. TRAIL mRNA and protein levels with 400 μg/mL UDCA treatment were only 2.42 and 5.30 times greater than control cells.

### 3.8. Gene Expression of NF-κB and IκB-α

**Figure 8:**
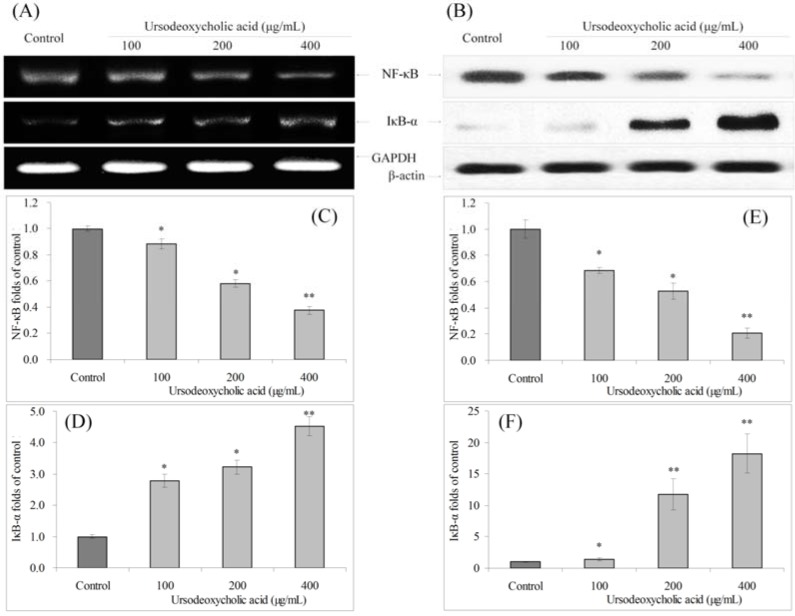
Effects of ursodeoxycholic acid (UDCA) on the mRNA (**A**) and protein (**B**) expression of NF-κB and IκB-α in human oral squamous carcinoma HSC-3 cells. Fold-ratio: gene expression/GAPDH (β-actin) × control numerical value (control fold ratio: 1); (**C**), NF-κB mRNA expression folds of control; (**D**), IκB-α mRNA expression folds of control; (**E**), NF-κB protein expression folds of control; and (**F**), IκB-α protein expression folds of control. * Mean values are significantly different from control (*p* < 0.05) and ** mean values are significantly different from control (*p* < 0.01).

UDCA could decrease the mRNA and protein expression of NF-κB and increase the expression of IκB-α compared to control cells (Figure 8).

## 4. Discussion

By evaluating the effect of UDCA on cancer and normal cells, it was seen that UDCA could significantly inhibit the growth of oral cancer cells *in vitro* and promote their death at and below 400 μg/mL, but it showed no obvious influence on normal oral cells at these concentrations. As a result, in this range of concentrations, UDCA was safe to normal oral cells and only affects cancer cells. UDCA showed inhibitory effects both in HSC-3 and TCA8813 cancer cells. The HSC-3 cell line was often used for oral science research [13,14,15] and was chosen for further experiments in this study. By using flow cytometry to detect cellular DNA content, it could be seen that the inhibitory effect of UDCA on cancer cell growth was in part due to promoting cancer cells into the sub-G1 phase and generating more apoptotic cells. Furthermore, evaluating the cells morphologically, UDCA could induce more cancer cell apoptosis visualized by DAPI staining.

GAPDH gene is stably and constitutively expressed at high expression levels in cancer cells and is used as a housekeeping gene. [16]. β-actin has relatively high and constant expression in the total protein of cancer cells. Therefore, β-actin has been widely employed in Western blotting as a loading control [17]. In this study, GAPDH and β-actin were also used as controls for RT-PCR and Western blot assays, respectively. The most important link in the mechanism of apoptosis is the activation of caspases. Caspases are proteases that participate in the control of cell growth and apoptosis [18]. Caspase-8 and caspase-9 are caspases upstream to the apoptosis signal transduction process, while caspase-3 is downstream, all of which are effector molecules of cell apoptosis [19]. Caspase-9 is an apoptosis effector molecule in the mitochondrial channel and starts the programmed cell death after activation. Subsequently, caspase-3 is activated, which amplifies and executes apoptotic signals and leads to apoptosis [20]. Studies have shown that with exogenous stimulation, the expression of caspase-3, caspase-8 and caspase-9 in *in vitro* cultured cancer cells were increased [11]. In a previous study, UDCA could also increase caspase-3, caspase-8 and caspase-9 activities to induce apoptosis in HepG2 hepatocellular carcinoma cells [21]. UDCA induced apoptosis through caspase-3 and also increased caspase-3 by decreasing Bcl-2 [22]. In this study, UDCA increased the mRNA and protein expressions of caspase-3, caspase-8 and caspase-9 in HSC-3 cells, signifying that UDCA treatment had anticancer effects through induction of caspases.

In apoptosis, the Bcl-2 family of proteins plays an important role because some of its members can promote apoptosis, such as Bax, while some members can prevent apoptosis, such as Bcl-2 and Bcl-xL [23]. Bcl-2 can prevent the release of cytochrome c (Cyt-c) from the mitochondria to the cytoplasm, thus inhibiting apoptosis. Bcl-2 is the most important member in the Bcl-2 family of proteins and regarded as an inhibitory effector of cell apoptosis [24]. In apoptosis signaling, Bcl-2 acts upstream of the activation of caspase-3 and inhibits its activation. Recent studies further showed that Bcl-2 not only acts upstream of caspase-3, but is also a direct substrate of caspase-3. After cleavage by caspase-3, the function of Bcl-2 completely changes from inhibition of apoptosis to promotion [25]. The Bcl-2 family of proteins is related to the caspase family of proteins in cancer cell apoptosis and treatment with UDCA increased Bax expression and reduced Bcl-2, Bcl-xL expression, thus possibly affecting the expressions of caspase-3, caspase-8, and caspase-9.

XIAP, the main member in the IAP family of proteins, is the strongest apoptosis inhibiting factor of the IAP family, and can directly inhibit caspases and regulate cell apoptosis in various ways. XIAP genes are expressed in most tumor cell lines, and its expression is closely related to tumor progression, recurrence, prognosis, and drug resistance to chemotherapy [26]. The main function of cIAP-1 is to mediate tumor necrosis factor receptor family for inhibition of apoptosis and decreasing apoptosis through direct interaction with caspase-3 [27]. Similarly, cIAP-2 is another kind of effective inhibitor of apoptosis by blocking the activity of caspase-9 induced by cytochrome c [28]. UDCA could increase the expression of caspases with XIAP, cIAP-1, and cIAP-2 decreased expression.

Survivin directly acts on caspases by inhibiting their activities and indirectly inhibiting caspases through p21. A combination of survivin and cell cycle regulation factor CDK4 can cause activation of CDK2/Cyclin-E and phosphorylation of ribosomes (Rb) [29]. Rb phosphorylation starts the cell cycle and speeds up transition between the G1/S phase, which causes p21 release from survivin-CDK4 complexes, which interacts with mitochondria pro-caspase-3, inhibiting the activity of caspase-3 and preventing release of Cyt-c from the mitochondria and ultimately inhibiting cell apoptosis [30]. UDCA could also reduce survivin expression, leading to increased caspases.

Fas is a death receptor and FasL is a product of T lymphocytes. Interaction between Fas and FasL can induce apoptosis [31]. Fas/FasL mediated apoptosis is the main pathway of cell apoptosis, transmitting apoptotic signals to caspases, activating a series of caspases to participate in apoptosis, and achieving self-regulation of caspases through cycle amplification and heterologous oligomerization [32]. Fas/FasL of HSC-3 oral cancer cells was changed with UDCA treatment. 

After combination of death receptors DR4, DR5 and TRAIL, oligomerization of death receptors occur and increases FADD molecules (Fas-associated death domain; also known as Mort1), which contain a DD (death domain) at the end of the *C*-terminus and a DED (death effector domain) at the *N*-terminus [33]. FADD can combine with receptors through the interaction between two DDs and increase the precursor of caspase-8, which is the initiator of apoptosis (pro-caspase-8), via the interaction between DED, and finally form the death signal complex DISC (death-inducing signaling complex). Within the DISC, the precursor of caspase-8 is activated to form caspase-8, thus causing apoptosis [34]. UDCA could increase DR4, DR5 and TRAIL, and this effect could promote caspase activation and induce apoptosis.

Defects in NF-κB increased susceptibility to apoptosis leading to increased cancer cells death. NF-κB regulates anti-apoptotic genes, especially the caspase family (generally speaking caspase family of proteins is not anti-apoptotic) [35]. Caspases could activate the NF-κB pathway independent of its activity as a pro-apoptotic protease. Death effector domains containing homologs of caspase-8 could similarly activate the NF-κB pathway. Dominant-negative mutants of the caspase-8 prodomain could block NF-κB induction by caspase-8 [36]. NF-κB is located in the cytosol complexed with the inhibitory protein IκB-α [37]. UDCA could increase IκB-α mRNA and protein expression and decrease NF-κB expression, aiding apoptosis.

## 5. Conclusions

As a monomeric material, UDCA has been introduced as treatment for various diseases, while the mechanism of the monomeric materials needs further research. By evaluating changes in gene and protein expression in cancer cells with UDCA treatment, we show that UDCA can be applied for the treatment of oral cancers. Preliminary *in vitro* experiments confirm that UDCA can induce cancer cell apoptosis by promoting caspase-3, caspase-8, caspase-9, Bax, Fas/FasL, TRAIL, DR4, DR5 and IκB-α gene expression in cancer cells, and reduce the expression of Bcl-2, Bcl-xL, XIAP, cIAP-1, cIAP-2, survivin and NF-κB.
